# A large-scale assessment of hand hygiene quality and the effectiveness of the “WHO 6-steps”

**DOI:** 10.1186/1471-2334-13-249

**Published:** 2013-05-30

**Authors:** László Szilágyi, Tamás Haidegger, Ákos Lehotsky, Melinda Nagy, Erik-Artur Csonka, Xiuying Sun, Kooi Li Ooi, Dale Fisher

**Affiliations:** 1 Department of Control Engineering and Information Technology (BME-IIT), Budapest University of Technology and Economics, Magyar tudósok krt. 2, Budapest H-1117, Hungary; 2 Faculty of Technical and Human Sciences, Sapientia University, Tîrgu Mureş, Romania; 3 Austrian Center for Medical Innovation and Technology (ACMIT), Wiener Neustadt,Austria; 4 Department of Nursing, National University Health System (NUHS), Singapore, Singapore; 5 Division of Infectious Diseases, University Medicine Cluster,National University Hospital, Singapore, Singapore; 6 Yong Loo Lin School of Medicine, National University Singapore, Singapore, Singapore

## Abstract

**Background:**

Hand hygiene compliance is generally assessed by observation of adherence to the “WHO five moments” using numbers of opportunities as the denominator. The quality of the activity is usually not monitored since there is no established methodology for the routine assessment of hand hygiene technique. The aim of this study was to objectively assess hand rub coverage of staff using a novel imaging technology and to look for patterns and trends in missed areas after the use of WHO’s 6 Step technique.

**Methods:**

A hand hygiene education and assessment program targeted 5200 clinical staff over 7 days at the National University Hospital, Singapore. Participants in small groups were guided by professional trainers through 5 educational stations, which included technique-training and UV light assessment supported by digital photography of hands. Objective criteria for satisfactory hand hygiene quality were defined a priori. The database of images created during the assessment program was analyzed subsequently. Patterns of poor hand hygiene quality were identified and linked to staff demographic.

**Results:**

Despite the assessment taking place immediately after the training, only 72% of staff achieved satisfactory coverage. Failure to adequately clean the dorsal and palmar aspects of the hand occurred in 24% and 18% of the instances, respectively. Fingertips were missed by 3.5*%* of subjects. The analysis based on 4642 records showed that nurses performed best (77% pass), and women performed better than men (75% vs. 62%, *p*<0.001). Further risk indicators have been identified regarding age and occupation.

**Conclusion:**

Ongoing education and training has a vital role in improving hand hygiene compliance and technique of clinical staff. Identification of typical sites of failure can help to develop improved training.

## Background

Failed hand hygiene of clinical staff is the major contributor to Healthcare-Associated Infections (HAIs) which occur in 7.1*%* of hospital admissions in the Western world [1]. Each year around 150,000 deaths in Europe and 100,000 in the USA are attributed to HAIs [2]. European and US standards have been developed in accordance with the World Health Organization’s (WHO) recommendations, defining 5 crucial moments of hand hygiene and 6 practical hand rubbing steps through which alcohol-based hand rub solutions have been proven effective [3].

Several studies have investigated staff compliance to hand hygiene guidelines, however, most involved relatively small number of subjects, typically 50–500 [4-8]. Studies of hand hygiene in hospitals have focused on hand washing standards [9-11], improving hand rubbing technique [12,13], or the composition of the hand rub and scrub [14-18], the attitude of staff regarding compliance to hand hygiene moments [19,20], the impact of finger rings, wrist watches and other accessories upon hand hygiene quality [8,21], and strategies of monitoring the compliance to the 5 moments [22]. Consensus guidelines and recommendations regarding hand hygiene have been produced through the WHO and descriptions on how they can be introduced have been published in [23,24]. Automated audits of hand hygiene have recently been trialled [25-27], but these are limited to monitoring compliance to the 5 moments rather than the quality of application as intended via the 6 steps.

The National University Hospital (NUH) of Singapore is a 1000-bed tertiary referral center. Hand hygiene training, assessment and monitoring have been a core activity since 2007, but the focus like in most other institutions has been on compliance with the 5 moments, which has improved from 15% to 69% based on up to 800 observations/month (unpublished). Less attention has been given to the technique and quality of hand hygiene [28].

In May 2011, NUH undertook a program to coincide with the WHO World Hand Hygiene Day within the frame of WHO Save Lives: Clean Your Hands campaign. Building from past subjective assessments of hand hygiene quality using Ultra Violet (UV)-marked alcoholic hand rub (Schülke Optik; Schülke & Mayr GmbH, Norderstedt, Germany) and regular desk top UV lights, we established a process for blinded assessment and storage of digital images for subsequent analysis. In this study digital images were used to analyze hand hygiene quality and therefore the effectiveness of a training program based on WHO’s 6 steps approach.

## Methods

### Procedure

Clinical staff received a 15-minute training in small groups (3–8 people), the first of which comprised of senior management, intentionally designed as a statement to encourage broad participation. In addition, staff were informed that undertaking the course was mandatory, with potential for personal financial penalties. As this was a regular educational exercise and the study was an audit of this process, ethics approval was not required.

At commencement of the training, all participants were registered and given information about the training and the evaluation. They received a personal Hand Hygiene Assessment Sheet (HHAS) where their progress was recorded. A stamp on the HHAS verified completion of each station. The programme consisted of 5 stations: 

•Station 1: Information on WHO’s 5 moments of hand hygiene.

•Station 2: Demonstration of WHO’s 6-step hand hygiene technique.

•Station 3: Individual hand hygiene practice with UV-marked hand rub solution.

•Station 4: Objective (double blinded) real-time assessment of hand hygiene technique with a purpose built imaging device.

•Station 5: Upon completion, a pledge recital and receiving of a sticker for their name tag certifying the credential.

Those who failed the assessment repeated the whole process.

The assessment of hand hygiene quality utilized the Stery-Hand monitoring devices provided by the Budapest University of Technology and Economics (BME, Hungary), and supported by a team from the developer group. Stery-Hand consisted of a black box with UV lighting inside and a digital camera connected to a notebook computer [29]. Digital images of both sides of employees’ hands were recorded. These images were then observed on separate screens, and evaluated by NUH infection control specialists, physically separated from the subjects. Pass and fail conditions had been determined, allowing a maximum of two small mistakes (see definition below) only on the dorsal side of the hand and no missed areas on the palmar aspect. We also investigated the distribution of “small” and “big” missed spots. According to the definition, every area uncovered by UV on either side of the hand was a mistake. Dark (untreated) spots >0.6 cm^2^ were defined as “big” mistakes. This tolerance margin allowed the faster and streamlined evaluation of the hand rubbing coverage.

All clinical staff of NUH (approx. 5200 people) were targeted for assessment. Images of participants’ hands were further processed and analyzed afterwards, creating a database on hospital-wide hand hygiene performance.

### Data collection and analysis

At Station 4 (assessment), each person was assigned an individual quick response (QR) code (ISO/IEC 18004:2006 standard) to facilitate anonymous identification. These codes were scanned and assigned to the respective images. The palmar and dorsal aspects of hands were recorded, along with personal data (age bracket, gender, occupation). Physically separated infection control team members could view the digital images on separate screens, quickly identify failings in the hand rub coverage with a basic (custom developed) drawing software, and transmit the results to the front-end trainers, who communicated them to the employee. The overall pass ratio according to the on-site “live” team was 67% (3108 out of 4642), despite the fact that trial and assessment took place immediately after demonstration. Thus 33% repeated the training, and were reassessed.

All data, original and evaluated images were stored. Only the images recorded at a participant’s first assessment have been included in this study.

### Statistical methods

The statistical analysis of the data was performed using the “R” program package, version 2.15.0 (The R Foundation for Statistical Computing, Vienna, Austria). The difference between two sets of samples was evaluated with 2-sample tests for equality of proportions with continuity correction, and considered significant if p-value was found less than 0.001. Confidence intervals were computed using the Wilson method [30].

## Results

Over 90% of eligible staff participated. Out of the 4762 enrolled staff members, 120 provided incomplete surveys, resulting in 4642 evaluable participants: 3559 females (age range 18–66 years, mean 35.2 years, standard deviation (SD) 10.0 years), and 1083 males (age range 18–73 years, mean 38.0 years, SD 11.1 years). Further breakdown of participants by age and occupation is shown in Table 1.

**Table 1:** Age and occupation distribution of the staff members participating in the assessment program

		**Age**									
**Occupation**	**Gender**	***−29***		**30–39**		**40–49**		***50+***		**Total**	
		**Subjects**	**Passed**	**Subjects**	**Passed**	**Subjects**	**Passed**	**Subjects**	**Passed**	**Subjects**	**Passed**
	Female	58	78%	100	73%	34	77%	20	85%	212	76%
Physicians	Male	46	74%	156	65%	78	76%	34	71%	314	70%
	All	104	76%	256	68%	112	76%	54	76%	526	72%
	Female	737	75%	594	80%	231	82%	141	80%	1703	78%
Nurses	Male	42	50%	25	64%	8	75%	8	63%	83	58%
	All	779	74%	619	80%	239	82%	149	79%	1786	77%
Environ-	Female	67	72%	78	67%	43	70%	69	67%	257	69%
mental	Male	44	64%	42	67%	23	35%	32	50%	141	57%
services	All	111	69%	120	67%	66	58%	101	61%	398	64%
Allied	Female	189	66%	134	68%	85	80%	72	79%	480	71%
health	Male	38	50%	56	73%	21	71%	20	60%	135	64%
	All	227	63%	190	70%	116	78%	92	75%	615	70%
	Female	364	72%	243	68%	167	77%	133	80%	907	73%
Others	Male	140	55%	111	53%	68	63%	91	69%	410	59%
	All	504	67%	354	63%	235	73%	224	76%	1317	69%
	Female	1415	73%	1149	75%	560	79%	435	78%	3559	75%
Total	Male	310	58%	390	63%	198	66%	185	65%	1083	62%
	All	1725	70%	1539	72%	758	76%	620	74%	4642	72%

Hand hygiene assessment results: number of passed, number of failed, and percentage of successful attempts out of the total number of participants, grouped by occupation and age range.

After the week-long assessment period, all images were reanalyzed applying the same definitions as used on site. This resulted in 3349 passes (72%) consistent with a good level of inter rater concordance between the on-site team and the later re-evaluation. The amount of overruled on-site decisions added up to approximately 8.5*%* of the total cases. The kappa concordance test between on-site evaluation and re-evaluation gave *κ*=0.80 (95% CI 0.78–0.82). We used the post-evaluation metrics for further analysis.

**Figure 1:**
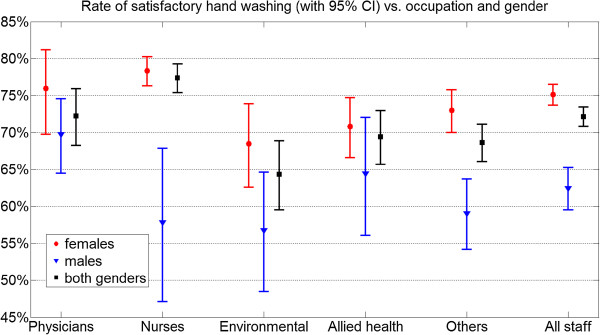
Satisfactory hand washing in various occupation groups: rates and confidence intervals.

**Figure 2:**
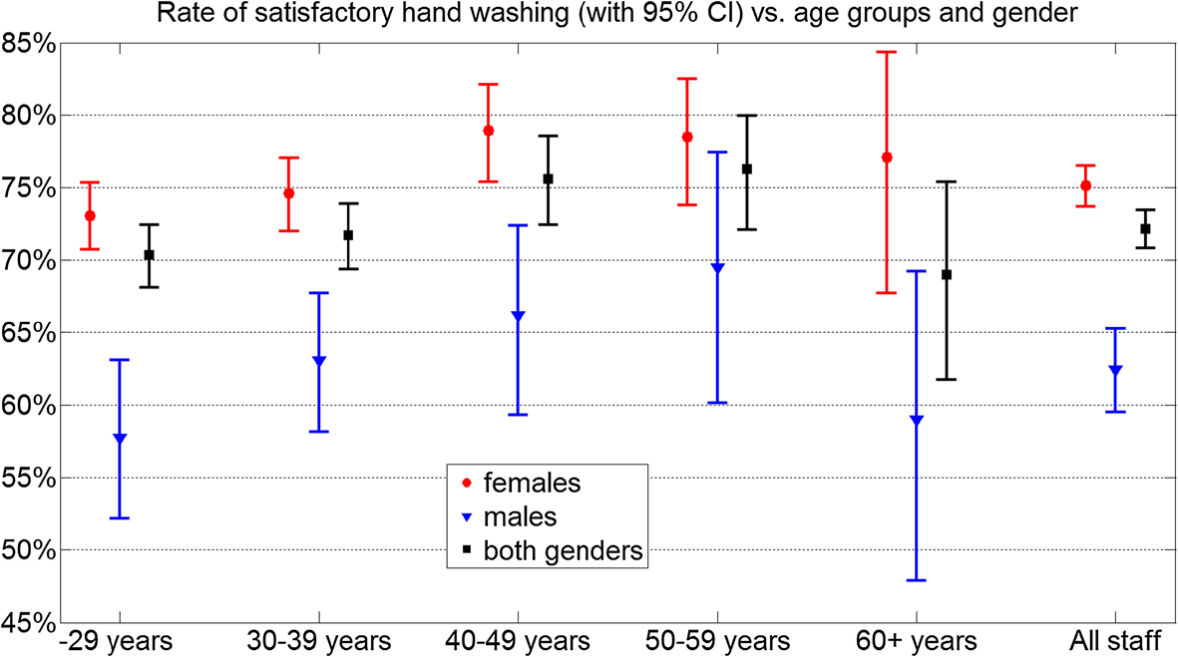
Satisfactory hand washing in various age ranges: rates and confidence intervals.

Pass rates by staff occupation type and demographics are summarized in Table 1. Figure 1 shows the rate and confidence interval of satisfactory hand washing, plotted against occupation and gender. Similarly, in Figure 2, rates and confidence intervals are arranged by age groups and gender.

The overall pass rate was 72% (95% CI 71%–73%). Female staff performed significantly better in almost all job and age categories, with an overall pass rate of 75% (95% CI 73%–77%) versus 62% (95% CI 59%–65%) that of males.

**Figure 3:**
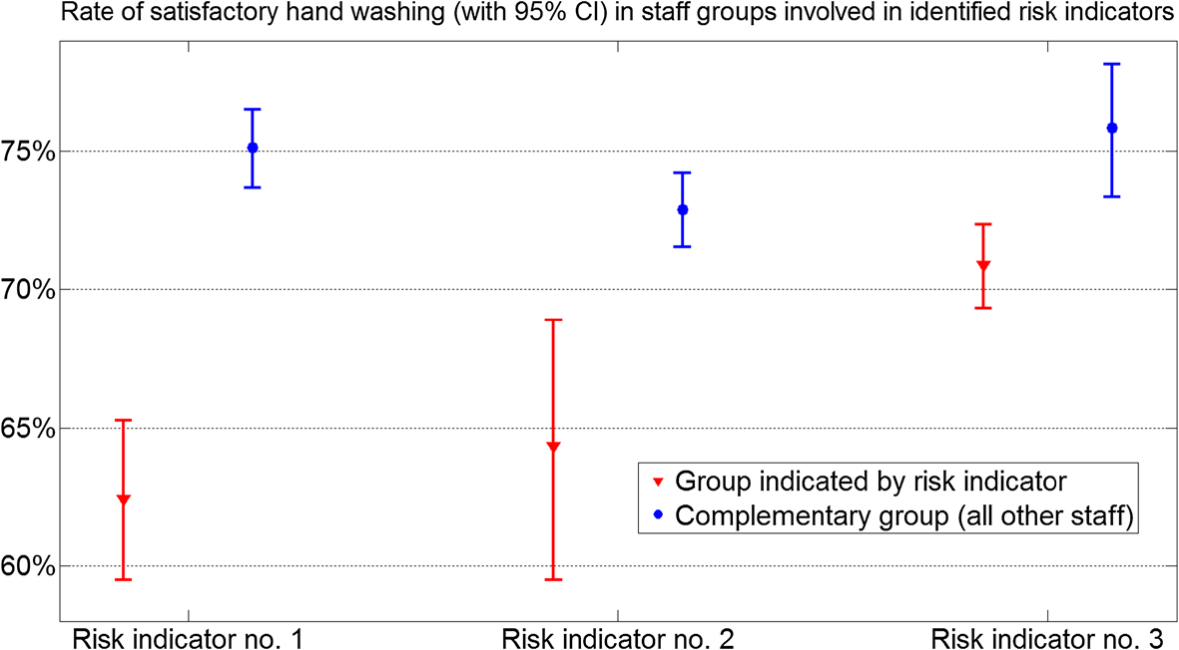
**Rates of adequate hand washing, with confidence intervals, in risk indicator groups and their complementary groups (Table **2).

Risk indicators (RI) for failed hand rub application were identified and listed in Table 2. For these risk groups, a higher incidence of unsatisfactory hand washing was found. Identified RI’s are supported by non-overlapping confidence intervals (Figure 3) and statistical significance.

**Table 2:** Identified risk indicators

**No.**	**Hypothesis tested**	**Incidence**	**Odds**	**Significance**
		**higher by**	**ratio**	
1	Male staff have higher incidence of unsatisfactory hand washing than females	51% (95% CI 32%–62%)	1.82 (95% CI 1.49–2.22)	*p*<0.001
2	Environmental service workers have higher incidence of unsatisfactory hand washing than all other job categories counted together	32% (95% CI 21%–45%)	1.49 (95% CI 1.14–1.96)	*p*<0.001
3	Staff aged –40 and 60+ have higher incidence of unsatisfactory hand washing than those aged 40–60	21% (95% CI 11%–30%)	1.29 (95% CI 1.05–1.58)	*p*=0.001

These risk indicators were found significant and relevant. Corresponding confidence intervals are shown in Figure 3.

**Figure 4:**
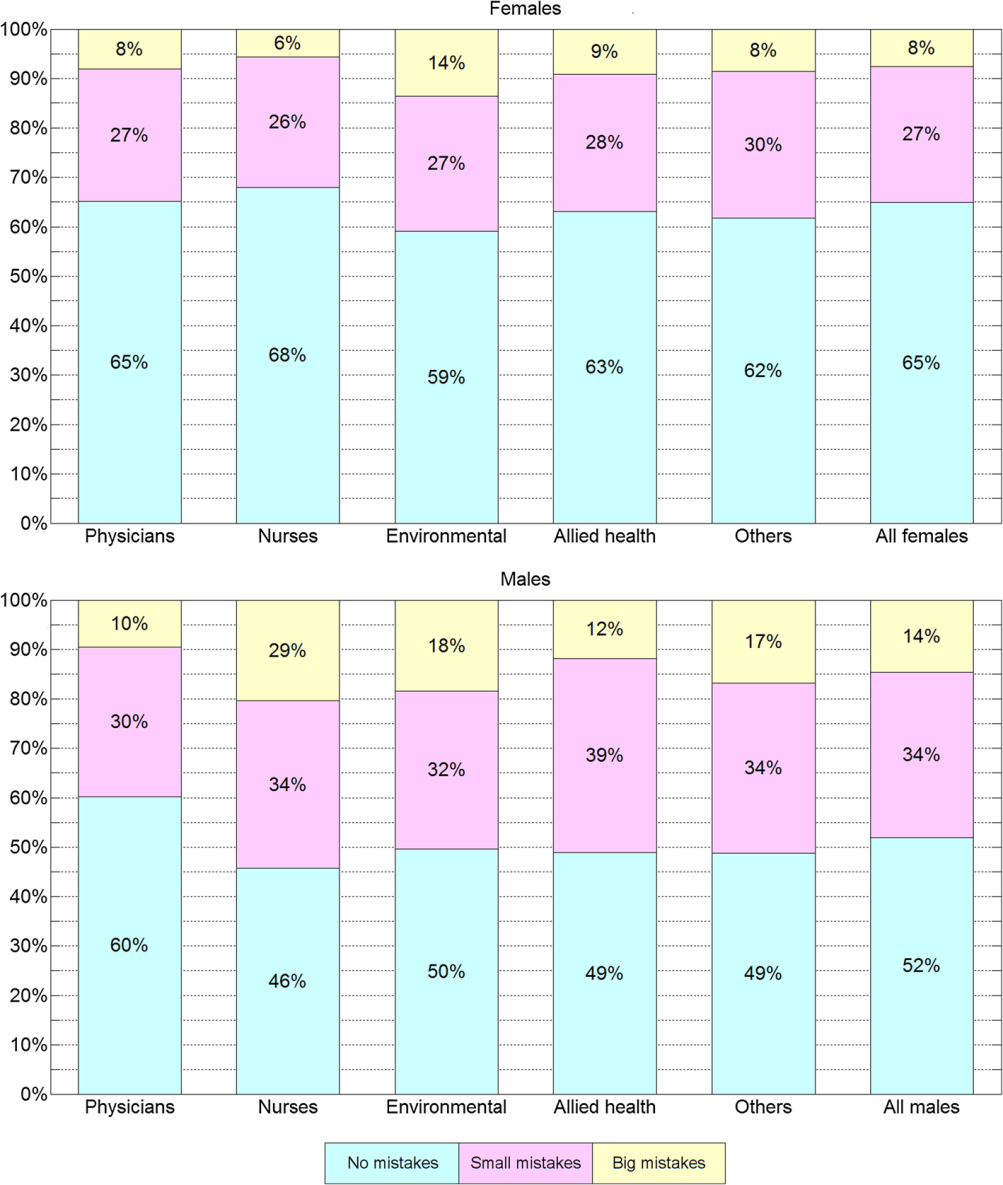
**Mistake location vs. gender and occupation.** Distribution of individuals with no mistakes, mistakes on the palmar side only, mistakes on the dorsal side only, and mistakes on both sides, respectively, grouped by gender and occupation.

**Figure 5:**
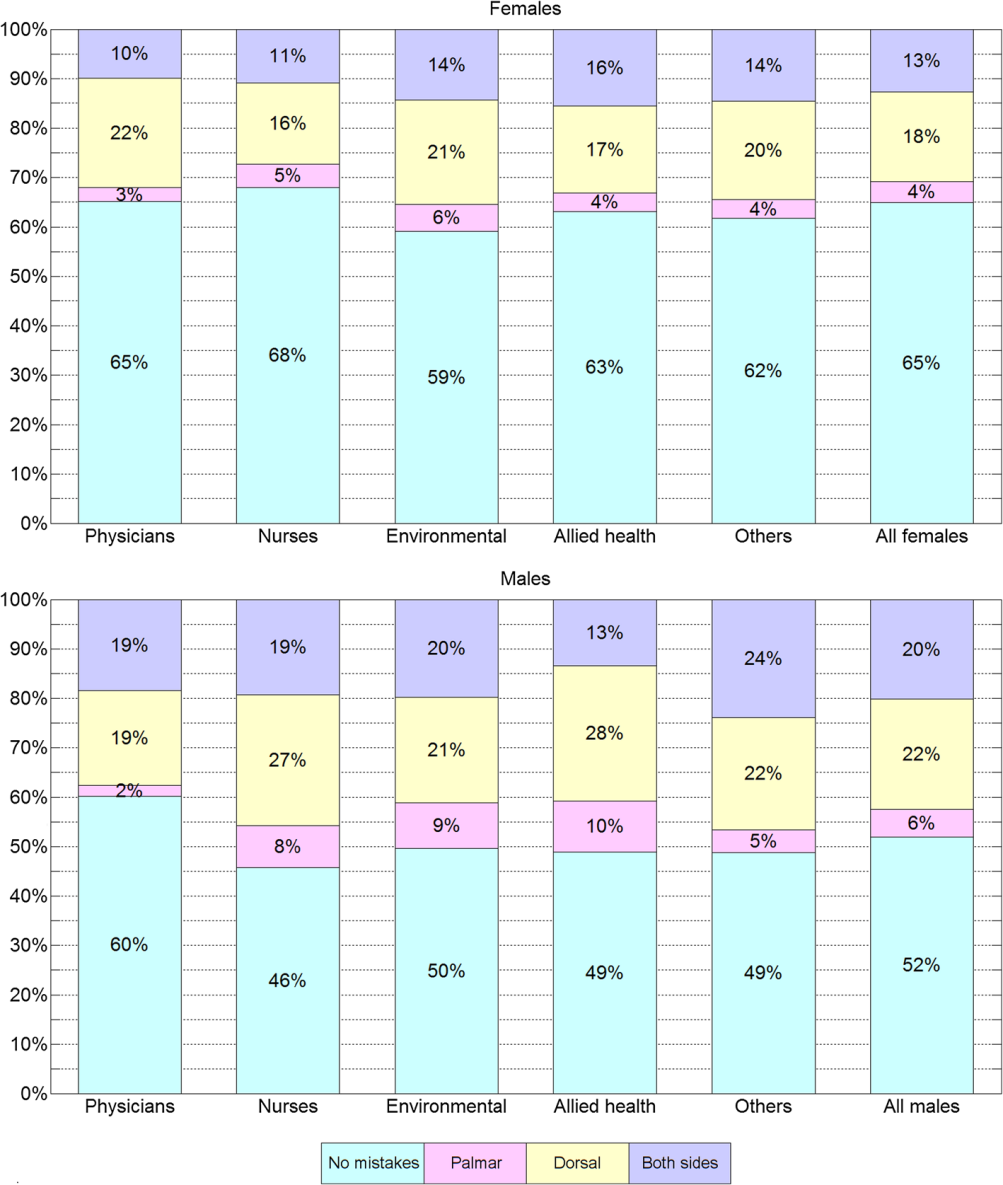
**Missed spot sizes vs. gender and occupation.** Distribution of individuals with no mistakes, small mistakes, and big mistakes, respectively, grouped by gender and occupation.

We classified all individuals into three quality groups based on whether they made no mistakes, small mistakes only, or big mistakes. Figure 4 shows the distribution of these groups defined by gender and occupation. Among those who failed the first assessment, we further distinguished three categories: those who made mistakes on the palmar side of the hand, on the dorsal side, or both. The distribution of these categories, within the gender and occupation groups is presented in Figure 5.

**Figure 6:**
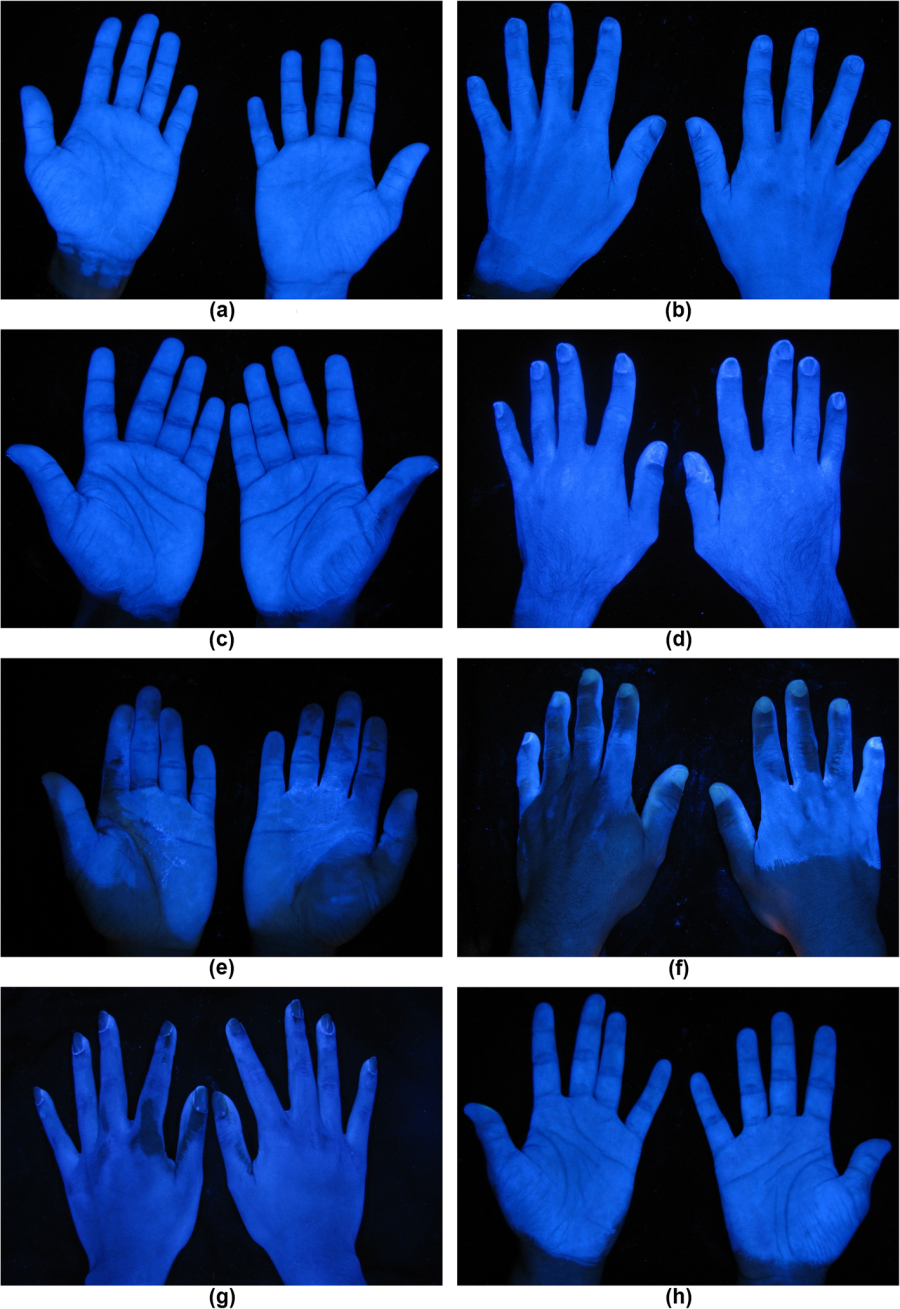
**Some examples of evaluated images.** Clean areas shine under UV light, dirty areas appear darker: (**a**)-(**b**) adequately cleaned hands; (**c**) small mistake on the palmar side; (**d**) small mistake on the dorsal side; (**e**) and (**f**) big mistakes on both sides; (**g**) mistakes in the area of fingers and thumbs; (**h**) mistake in the proximity of wrist crease.

The most frequently missed sites were identified on the dorsal side of fingers in the proximity of the nails (16%), on the thenar eminence and in the proximity of the wrist crease (18%). Figure 6 shows example images taken during the exercise.

## Discussion

This study evaluated the effectiveness of the WHO 6-step hand rubbing technique, involving a large number of health care workers and a novel screening technology.

A key advantage of the applied methodology is the ability to evaluate the actual outcome of hand washing and in this situation, the educational process. Identifying failures in hand hygiene coverage represents an opportunity to enhance one’s infection prevention efforts. Identifying certain risk groups and typical mistakes regarding the application patterns allows for the design of specific education programs and also helps to left the use of a hand rubbing protocol. Another advantage of the methodology is the ability to easily involve several thousand health care staff in the investigation, a major increase compared to the 465 subjects of the largest similar study [8]. Instead of applying restricted observation of nurses and physicians only, as most similar studies do, our investigations involved all staff of a single hospital because all health workers have the potential to contribute to cross-infection [31].

Previous studies have found a higher incidence of unsatisfactory hand hygiene in males, but this difference is generally insignificant possibly due to small samples size. A recent study culturing glove juice found the prevalence of Enterobacteriaceae higher in males by 55% (23.8% vs. 15.4%, *p*=0.156) [8], similar to our findings (Table 2). The differences we found between various occupational groups were similar to non-significant trends observed by others [32-34], but in our study statistical significance was evident.

Our study did not apply microbiological validation but assumed that bright areas of the hand were clean, while dark spots could contain potentially dangerous pathogens. The general intention in hand hygiene is to fully cover the surface of both hands with antiseptic solution. Our methodology monitors this information. Microbiological techniques provide an alternative and arguably more useful assessment but are more complex and expensive to undertake particularly on this scale. All commercial solutions must undergo specific tests for efficacy against germs: they are approved as bactericidal (EN 1276), fungicidal (EN 1650), or sporicidal (EN 13704) solutions. These tests provide the evidence that once the surface is treated appropriately, efficacy is guaranteed. Occasionally microbiological studies have been employed for randomized hand hygiene assessment, however, always on a small scale [5,8]. Nevertheless, sampling and cultivation are susceptible to environmental effects and cross-contamination, distorting the results of the evaluation. In this respect our methodology provides a simpler alternative to culture techniques.

The main contribution of our study is not the monitoring methodology itself, but the statistical findings of the hand hygiene quality assessment. Microbiological validation of our monitoring and assessment methodology is an area for future research.

The setting of our study is likely to have actually overestimated the real life quality of hand rub application given that it was a specific and overtly observed assessment. The generalizability of our findings therefore has some limitations however monitoring staff in everyday practice would carry significant challenges. The fact that a single measurement was taken of each subject, and the deviation between on-site and subsequent evaluation, may slightly distort the statistical data.Similarly, another limitation of the study is that it was undertaken at a single institution.

This study has demonstrated that a high failure rate in hand hygiene coverage among health care workers can occur even when observed immediately after a specific training programme of the WHO’s 6 steps. It is a significant and generally underrated risk factor since imperfect hand hygiene technique may endanger the effectiveness of the entire hand hygiene routine.

## Conclusion

We established an environment to observe and evaluate the effectiveness of the 6 step hand washing procedure, which—when applied at the times of the WHO 5 moments—is considered the most effective way to prevent the transmission of HAIs. The quality of hand rub application was analyzed on 4642 hospital staff immediately after a personalized education program. High failure rates suggest that the 6 steps, as advocated by the WHO, may be too complex to expect staff to comply with routinely. Our findings have revealed that besides effective monitoring of compliance with the five moments, there is a strong need for improved, targeted educational efforts on handhygiene technique.

## Abbreviations

UV: Ultraviolet; HAI: Health care-associated infections.

## Competing interest

TH, MN, ÁL and LS are listed as inventors on a pending patent related to hand hygiene control: “Method and apparatus for hand disinfection quality control”, (Budapest University of Technology and Economics) PCT/HU2011/000094, WO2012042285, 2010.

## Authors’ contributions

ÁL, MN, LS and TH designed and implemented the measurement system. DF, TH, MN, LS, KLO and XS designed the assessment process. All authors participated in the data collection. DF, KLO and XS were responsible for on-site expert evaluation. ÁL, MN, LS, TH and EAC performed the re-evaluation of the collected data. LS and TH were responsible for the statistical data analysis. LS, TH and DF prepared the manuscript. All authors read and approved the final version of the manuscript before submission.

## Pre-publication history

The pre-publication history for this paper can be accessed here:

http://www.biomedcentral.com/1471-2334/13/249/prepub
